# The cognitive and neural underpinnings of discourse coherence in post-stroke aphasia

**DOI:** 10.1093/braincomms/fcac147

**Published:** 2022-06-14

**Authors:** Reem S W Alyahya, Matthew A Lambon Ralph, Ajay Halai, Paul Hoffman

**Affiliations:** King Fahad Medical City, Riyadh, Saudi Arabia; MRC Cognition and Brain Sciences Unit, University of Cambridge, Cambridge, UK; Faculty of Medicine, Alfaisal University, Saudi Arabia; Division of Language and Communication Science, School of Health Sciences, City University, London, UK; MRC Cognition and Brain Sciences Unit, University of Cambridge, Cambridge, UK; MRC Cognition and Brain Sciences Unit, University of Cambridge, Cambridge, UK; School of Philosophy, Psychology & Language Sciences, University of Edinburgh, Edinburgh, UK

**Keywords:** aphasia, discourse coherence, lesion-symptom mapping, prefrontal cortex, executive functions

## Abstract

Although impaired discourse production is one of the prominent features of aphasia, only a handful of investigations have addressed the cognitive, linguistic and neural processes that support the production of coherent discourse. In this study, we investigated the cognitive and neural correlates of discourse coherence in a large mixed cohort of patients with post-stroke aphasia, including the first voxel-based lesion-symptom mapping of coherence deficits. Discourse responses using different tasks were collected from 46 patients with post-stroke aphasia, including a wide range of classifications and severity levels, and 20 matched neuro-typical controls. Global coherence, defined as the degree to which utterances related to the expected topic of discourse, was estimated using a previously validated computational linguistic approach. Coherence was then related to fundamental language and cognitive components in aphasia identified using an extensive neuropsychological battery. Relative to neuro-typical controls, patients with aphasia exhibited impaired coherence, and their ability to maintain coherent discourse was related to their performance on other language components: phonological production, fluency and semantic processing, rather than executive functions or motor speech. These results suggest that impairments in core language components play a role in reducing discourse coherence in post-stroke aphasia. Whole-brain voxel-wise lesion-symptom mapping using univariate and multivariate approaches identified the contribution of the left prefrontal cortex, and particularly the inferior frontal gyrus (pars triangularis), to discourse coherence. These findings provide convergent evidence for the role of the inferior frontal gyrus in maintaining discourse coherence, which is consistent with the established role of this region in producing connected speech and semantic control (organizing and selecting appropriate context-relevant concepts). These results make an important contribution to understanding the root causes of disrupted discourse production in post-stroke aphasia.

## Teaser

Alyahya *et al*. found discourse coherence deficits in post-stroke aphasia to be related to impairments in general language components (phonological production, fluency and semantic processing) rather than executive functions. They found an association between discourse coherence deficits and lesions to the left prefrontal cortex, particularly the inferior frontal gyrus (pars triangularis).

## Graphical Abstract

**Graphical abstract:**
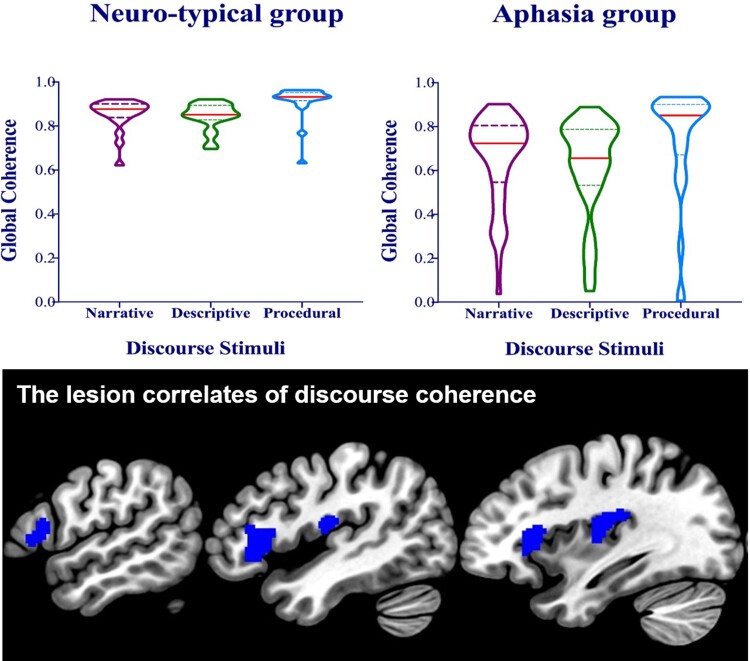

## Introduction

To communicate effectively in conversation, speakers must produce statements that are informative and relevant to the topic under discussion. Speech that meets these challenges is said to be coherent. Coherent discourse involves a series of well-linked utterances that are related to each other and to the specific topic of conversation.^1–3^ In this study, we focus specifically on global coherence, which reflects the degree to which each statement of the discourse relates to the current topic of conversation.^1,2^ Producing coherent discourse is often impaired following brain damage or mental disorders, including right hemisphere stroke,^4,5^ schizophrenia,^6^ traumatic brain injury^7,8^ and Alzheimer’s disease.^7^ Although impaired coherence might hinder effective communication, the cognitive and neural correlates of coherence in post-stroke aphasia remain uncertain. This is the focus of the present article.

The literature is inconsistent with regards to coherence deficits in people with post-stroke aphasia (i.e. an acquired language disorder that typically presents with deficits in discourse production). Previous studies that focused on fluent types of aphasia have found impaired global coherence post-stroke in patients with co-existing impaired linguistic processes at the phonological, lexical and syntactic levels;^9–12^ however, they typically contain small samples. A few studies, in contrast, have reported intact global coherence in people with fluent aphasia.^7,13^ This discrepancy in the literature might relate to several factors that have differed across studies. First, the majority of studies tested people with fluent aphasia but not those with less fluent aphasia.^7,10,11,14^ Second, discourse elicitation tasks vary across investigations and individual studies have typically only utilized a single type of discourse task, such as picture description (e.g.^6,10,15^), storytelling narratives (e.g.^9,11–13^), procedural discourse (e.g. ^9,14^) or personal discourse (e.g.^4,7,16,17^). Different discourse tasks can lead to variations in connected speech and discourse features in aphasia, including production quantity,^18–21^ lexical and semantic features,^22^ and coherence.^15,23^

The mechanisms that support connected speech production can be divided into two sets of interconnected processes: microlinguistic, which focuses on phonological, lexical and syntactic processes; and macrolinguistic, which refers to processes that organize and regulate the content and topic of speech.^1,2,7^ Deficits at either level of processing can result in coherence deficits in different patient groups; however, the root causes of impaired coherence in post-stroke aphasia remain indeterminate. Some researchers have proposed that coherence deficits in aphasia are primarily caused by disruptions to language skills at the microlinguistic level. Specifically, it has been shown that lexical diversity can predict the level of global coherence in aphasia,^12^ that word retrieval deficits are related to reduced coherence,^10^ and that reduced information content of a discourse contributes to impaired global coherence in people with aphasia and healthy controls.^12,14^ Thus, inability to access particular words and concepts may limits a patient’s ability to produce utterances that conform to the topic of conversation. In addition, it has been argued that people with aphasia tend to use adaptive strategies, such as repetitions, preservations and fillers to overcome their expressive language deficits, which might reduce the level of coherence.^10,11^ An alternative view holds that impairments to broader cognitive processes in patients with aphasia, including attention, episodic memory and executive control, disrupt the coherence of their speech.^1,4,8,15,17^ This view is supported by the reduced coherence observed in non-aphasic patient groups who present with deficits in these domains, such as those with right hemisphere stroke,^4,5^ traumatic brain injury,^7,8^ Alzheimer’s disease^7^ and schizophrenia.^6^

No studies to date have systematically investigated the lesion correlates of coherence deficits in stroke patients. Such investigations are critical to gain insights into the root causes of coherence deficits following stroke. Across the broader neuroimaging literature, relatively few studies have investigated the neural underpinnings of connected speech production, including coherence. A wide left-lateralized network supporting connected speech production has been identified in functional neuroimaging experiments^24–26^ and lesion-symptom mapping studies.^18,22,27^ This network involves the prefrontal cortex, including regions implicated with motor planning, fluency, and cognitive control, as well as regions in the anterior and posterior temporal lobe and posterior parietal lobe, which have been associated with the representation of semantic knowledge. More specifically, the role of the left prefrontal cortex has been highlighted in association with maintaining appropriate and informative connected speech in post-stroke aphasia,^18^ and damage to the left inferior frontal gyrus (IFG) and insula were associated with impaired lexical selection.^27,28^ A recent fMRI experiment with healthy older adults found that frontal activation in bilateral IFG predicted global coherence in speech production.^29^ This supported the view that the inability to inhibit irrelevant information contributes to reduced global coherence,^17^ as the IFG has been implicated in this function both in healthy participants^30,31^ and patients.^32^ A neuro-stimulation experiment also reported impaired global coherence with no changes to verbal productivity following transient suppression of the left IFG in healthy adults.^33^ Findings from these studies highlight the role of the left prefrontal cortex in regulating conceptual organization and selection of an appropriate message. It is unclear, however, whether lesions to this area contribute to poor coherence in stroke aphasia.

In the current study, we initially examined whether patients with post-stroke aphasia would exhibit impaired coherence, relative to neuro-typical adults. This was assessed using three different discourse tasks (picture description, storytelling narrative, and procedural discourse) across the two groups to account for variations resulting from the elicitation method. Second, we investigated whether coherence deficits in post-stroke aphasia can be predicted by deficits to other aspects of language and executive functions. Finally, and for the first time, we identified the lesion correlates of coherence using whole-brain voxel-wise lesion-symptom mapping. We used both univariate and multivariate approaches as previous studies have shown that using both approaches can be complementary to one another.^34,35^ Generally, univariate analyses assign beta values to voxels in a relatively transparent way, in which the strength and sign of these values indicate meaningful brain-behaviour relationships. This allows for easy interpretation of each weight associated with a voxel. On the other hand, multivariate approaches have the potential bonus of revealing more complex relationships between behavioural variation and multiple voxels.^36,37^ To shed light into the cognitive and neural mechanisms of coherence across the whole range of the aphasia spectrum, patient recruitment was not limited to a particular aphasia classification or severity level. Moreover, this is the largest investigation of coherence in post-stroke aphasia to date, not only in terms of sample size but also due to the detailed neuropsychological background data available and the use of lesion-symptom mapping approaches.

## Methods

### Participants

Forty-six patients who had developed aphasia following a single left haemorrhagic or ischaemic stroke were tested in the chronic stage (>12 months post-stroke). This is the same cohort who had participated in our previous study.^18^ Aphasia was diagnosed and classified using the Boston Diagnostic Aphasia Examination (BDAE^38^). All patients were native English-speakers with normal or corrected-to-normal vision and hearing. The exclusion criteria included multiple strokes or any other neurological conditions, severe motor speech disorders, any contraindications for MRI scanning, being pre-morbidly left-handed and patients who did not produce any response in all discourse tasks. In addition to the patient group, discourse samples were collected from the age of 20/education-matched neuro-typical controls. They were native English-speakers, right handed and reported no abnormal neurological conditions or history of brain injury. Demographic information for both groups is presented in Table 1. Informed consent was obtained from all participants before participation under approval from the local ethics committee.

**Table 1:** Participant’s demographic information

Demographic variables	Control group (*N* = 20)	Aphasia group (*N* = 46)
**Gender:** Male:female ratio	9:11	32:14
**Age:** Mean (range, SD)	68.85 years (57–84, 8.47)	63.21 years (44–87, 11.93)
**Education:** Mean (range, SD)	14 years (9–19, 2.8)	12.65 years (9–19, 2.59)
**Time post-stroke onset:** Mean months (range, SD)	N/A	69.43 months (16–280, 48.86)
**Lesion size (voxel):** Mean (range, SD)	N/A	15497 (175–41379, 11188)
**Aphasia severity** ^a^: Mean (range, SD)	N/A	2.8 (1–5, 1.2)
**Aphasia classification** ^a^:	N/A	*Fluent aphasia*:
		Anomia = 20
		Conduction = 4
		Transcortical Sensory = 1
		*Non-fluent aphasia*:
		Transcortical Mixed = 1
		Broca's = 9
		Mixed non-fluent = 8
		Global = 3

^a^ Aphasia severity and classification were determined using the Boston Diagnostic Aphasia Examination.^35^

### Discourse samples: elicitation, transcription and computation of coherence

Three discourse samples were collected from each participant with no time limit: (i) descriptive discourse, elicited using the ‘Cookie Theft’ composite picture from the BDAE;^38^ (ii) narrative discourse, elicited using a ‘Dinner Party’ storytelling script,^39^ which involved a series of eight black-and-white sequences of pictures; and (iii) procedural discourse, which was elicited by asking participants to describe ‘how they prepare a cup of tea’. No prompts or questions were provided except for non-verbal encouragement. In the first two discourse tasks, participants were presented with the picture stimuli and were asked to look through them and then describe in detail what was going on in these pictures. Each sample was digitally recorded, transcribed verbatim, followed by content analyses completed by the first author (R.S.W.A.), a qualified and experienced speech and language pathologist.

Global coherence was measured using an objective automated computational linguistic approach, first described by Hoffman, Loginova, Russell,^16^ implemented in R, using publicly available code (https://osf.io/8atfn/). The approach utilized latent semantic analysis (LSA),^40^ which provides vector-based representations of the semantic content of each discourse sample. These can be analysed to determine the degree to which the semantic content of a particular discourse sample conforms to the expected topic, given the stimulus or prompt. It has been shown that this approach to computing coherence has high internal reliability and test–retest reliability, and it is highly correlated with subjective ratings of coherence.^16^

We used discourse samples from the neuro-typical group to determine the typical semantic content expected in response to each discourse stimulus. To do this, we computed an LSA vector representation for each control participant’s response and averaged these to provide a composite vector that represented the prototypical semantic content produced by healthy individuals in response to each discourse stimulus. All word types except functional words contributed to the composite vector (including nouns, verbs, adjectives and adverbs). Global coherence for each patient per stimulus was computed by taking the LSA vector for the patient’s entire response and comparing this with the composite representation for the same stimulus. Global coherence was defined as the similarity (measured using cosine) between the patient’s vector value and the composite vector. Therefore, this coherence measure captures the degree to which a patient’s speech conforms to the topics expected in response to the presented stimulus, in accordance with the definition of global coherence.^1,2^ A low value would be obtained if a patient had a tendency to talk about other topics or elements not related to the stimulus. Global coherence values were computed for the control participants in the same fashion, except that, to ensure independence, a participant’s own response was excluded when computing the composite vector. Further details of the LSA space and the averaging procedure are reported by Hoffman, Loginova, Russell.^16^ The only difference between the procedure used here and previous implementations of the coherence method^16,17,29^ was that here we used one vector to represent the entire patient response rather than dividing the response into a number of smaller windows or chunks. We did this because some patients gave short responses which could not be sub-divided into smaller windows.

### Acquisition and processing of neuroimaging data

High-resolution structural T_1_-weighted MRI scans were acquired for each patient on a 3.0T Philips Achieva scanner (Philips Healthcare, Best, The Netherlands) using an eight-element SENSE head coil. A T_1_-weighted inversion recovery sequence with 3D acquisition was utilized with the following parameters: repetition time = 9.0 ms, echo time = 3.93 ms, acquired voxel size = 1.0 × 1.0 × 1.0 mm^3^, slice thickness = 1 mm, matrix size = 256 x 256, 150 contiguous slices, flip angle = 8, field of view = 256 mm, inversion time = 1150 ms, SENSE acceleration factor 2.5, total scan acquisition time = 575 s.

Participants’ structural T_1_-weighted MRI scans were pre-processed and analysed with Statistical Parametric Mapping software (SPM12: Wellcome Trust Centre for Neuroimaging, http://www.fil.ion.ucl.ac.uk/spm/) running under Matlab (R2018a). The images were normalized into standard Montreal Neurological Institute (MNI) space using a modified unified segmentation-normalization tool optimized for focal brain lesions.^41^ Structural scans from an age- and education-matched control group (18 male and 4 female; mean age = 69.13 years, SD = 5.85; and mean years of education = 13 years, SD = 2.66) were used as reference to identify abnormal tissue in the stroke group using a fuzzy clustering fixed prototypes (FCP) approach. This produces a whole-brain map where each voxel is a probability of abnormality compared with the control group. We applied a binary threshold to this image to obtain a binary lesion image (i.e. U-threshold = 0.5). The images generated for each patient were visually inspected with respect to the original scan and manually corrected if necessary and were used to generate a lesion overlap map. The fuzzy images were then smoothed with an 8 mm full width half maximum Gaussian kernel, to account for the global intra-individual shape differences.

### Statistical analyses

Initially, to examine the differences in discourse coherence between groups and across discourse tasks, we conducted a 2 × 3 mixed ANOVA with global coherence as the dependent variable, group (controls versus patients with aphasia) as the between-subject factor and discourse tasks (composite picture description versus storytelling narrative versus procedural) as the within-subject factor.

To investigate the degree to which coherence deficits in post-stroke aphasia can be attributed to different language and/or cognitive components, we utilized the fundamental language and cognitive components in aphasia that were identified in a previous study on the same patient group.^18^ To obtain these components, we administered a multivariate data-reduction technique (principal component analysis) on an extensive neuropsychological battery that consisted of tests of phonemic discrimination, comprehension at the word and sentence levels, semantic processing, repetition, naming, working memory, executive functions, and several discourse measures extracted from different discourse responses, including content word count, lexical diversity, informativeness, and words-per-minute. The principal component analysis generated seven orthogonal components including three connected speech ones reflecting verbal fluency, verbal quality, and motor speech, alongside four core components relating to phonological production, semantic processing, phonological recognition, and executive functions. Specifically, (i) picture naming and delayed and immediate repetition tests loaded on the ‘phonological production’ component; (ii) content word count measures from all discourse samples loaded on the ‘verbal fluency’ component; (iii) the ‘semantic processing’ component loaded with comprehension and production tests that probe semantic knowledge, including the spoken sentence comprehension test from the comprehensive aphasia test;^42^ 96-synonym judgement test;^43^ noun and verb picture-to-word matching;^44^ verb synonym judgement test;^45^ spoken and written word-to-picture matching, Camel and Cactus test, and Cambridge naming test from the Cambridge Semantic Battery;^46^ Boston Naming Test;^47^ and Object and Action Naming Battery;^48^ (iv) the ‘verbal quality’ component loaded with informativeness measures from all discourse samples, which represent the accuracy of the provided information; (v) phonemic discrimination tests loaded on the ‘phonological recognition’ component; (vi) the ‘motor speech’ component loaded with speech rate measure from all discourse samples; and (vii) the non-verbal executive tests (i.e. Brixton Spatial Rule Anticipation Task^49^ and Raven’s Coloured Progressive Matrices^50^) loaded on the ‘executive function’ component. For further details on the computation of connected speech measures and the statistical method, see Alyahya *et al*.^18^

In the present study, we created a series of simultaneous multiple regression models, with a composite global coherence score (computed by averaging the global coherence values across the three discourse tasks) used as the dependent variable. Patients’ component scores on the seven language and cognitive components (as described above) were used as the independent variables in stages, as follows: Model 1 included main language components (phonological production, phonological recognition and semantic processing); Model 2 included the connected speech components (verbal fluency, verbal quality and motor speech) in addition to the variables included in Model 1; Model 3 included the executive functions component in addition to the variables included in Model 2; In Model 4, we added interaction terms between executive functions and the significant variables identified from the first three Models, in addition to the variables included in Model 3. These models were created to test whether the additional components would improve the model fit and would explain further variance associated with discourse coherence in aphasia.

Finally, to identify the lesion correlates associated with coherence deficits, we conducted both univariate and multivariate lesion-symptom mapping analyses. We used voxel-based correlational methodology (VBCM^51^), an approach that identifies statistical relationships between brain and behaviour by correlating the value per voxel (as a continuous variable) with the behavioural performance. We created multiple regression models on the FCP whole-brain images (% abnormality) with composite discourse coherence scores entered as a regressor of interest and demographic variables (age and months post-stroke onset) entered as covariates. Lesion volume (estimated using the automated lesion identification tool^41^) was entered as a covariate in a subsequent analysis. Two analyses (with/without lesion volume covariate) were performed to avoid a possible risk for Type II error with the inclusion of lesion volume. It had been argued that because lesion volume has a non-trivial relationship with anatomy,^52,53^ some brain regions showing strong correlations with lesion volume would be penalised while others will be favoured once lesion volume is included in the lesion mapping analysis, resulting in potentially increased distortions.^54^ Therefore, we present both models, with and without lesion volume correction. The results were thresholded at *P* < 0.001 voxel-level and cluster-corrected using family-wise error (FWE) at *P* < 0.05. The multivariate analysis was conducted to supplement the univariate analysis, using support-vector regression lesion-symptom mapping (SVR-LSM) toolbox.^55^ The binary lesion images were loaded as the features in the model, and the composite coherence scores were used as the measure of interest, with demographic variables (age and months post-stroke onset) entered as covariates, using MATLAB’s SVR procedure with the following settings: MATLAB SVR implementation, hyper-parameter optimization (Bayes optimization with default settings) and lesion threshold = 4. As with the VBCM, we ran the model again where lesion volume was included as a covariate on both lesion and behavioural data as recommended by DeMarco, Turkeltuab.^55^ The resulting support-vector regression beta weights were thresholded at voxel-wise *P* < 0.005 and corrected for cluster size at *P* < 0.05, both based on 100 00 permutations.

### Data availability statement

The code to compute coherence is available in the Open Science Foundation repository, https://osf.io/8atfn/. The data necessary for reproducing the results in this paper can be provided upon reasonable request from the corresponding authors.

## Results

### Differences in discourse coherence between patients with aphasia and neuro-typical controls

**Figure 1:**
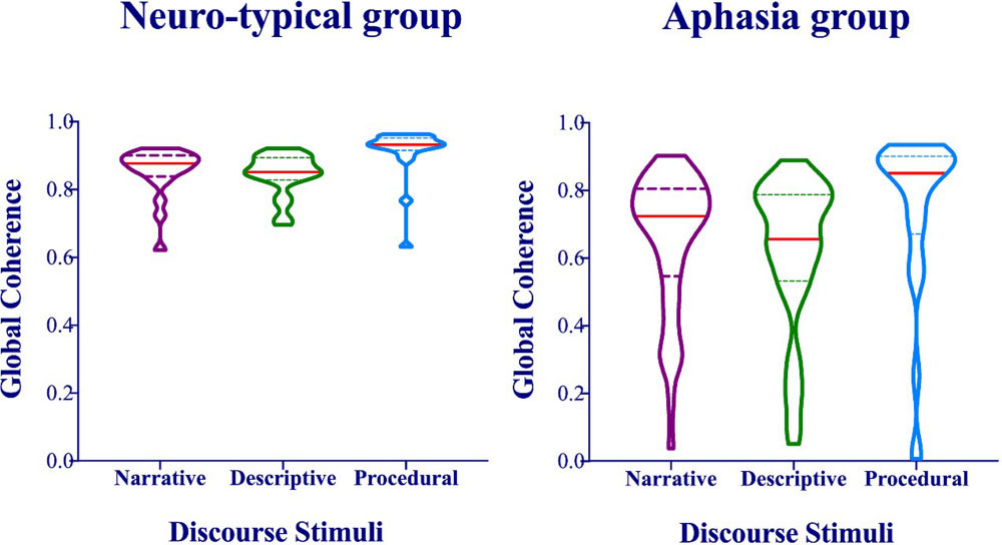
**Discourse coherence by patients with aphasia and neuro-typical controls.** Violin plots showing the distribution of data and the probability density of discourse coherence produced during three discourse tasks among groups of neuro-typical adults (*N* = 20) and patients with aphasia (*N* = 46). Straight red lines refer to the group median, top dotted lines refer to the third quartile, and bottom dotted lines refer to the first quartile. The differences between the two groups on each discourse task was statistically significant (*P* < 0.001)

The distribution of the discourse coherence data is illustrated in Fig. 1 and descriptive statistics are reported in Table 2. The ANOVA revealed a significant group effect (*F*(1,64) = 18.44, *P* < 0.001, partial *η^2^* = 0.23) with higher coherence scores among the control group compared to the patient group. A significant main effect of discourse tasks was also established (*F*(2,128) = 12.22, *P* < 0.001, partial *η^2^* = 0.16), with significantly higher coherence during procedural discourse compared with both storytelling narrative and picture description (*P* ≤ 0.001). However, the group × discourse interaction was not significant.

**Table 2:** Descriptive statistics of global coherence produced by the control and aphasia groups

	Control group (*N* = 20)		Aphasia group (*N* = 46)		
Discourse	Mean	SD	Mean	SD	#Patients with coherence deficits^a^
Storytelling narrative	**0**.**86**	**0**.**07**	**0**.**65**	**0**.**20**	**30**
Picture description	**0**.**84**	**0**.**06**	**0**.**61**	**0**.**22**	**30**
Procedural discourse	**0**.**91**	**0**.**07**	**0**.**74**	**0**.**25**	**18**

^a^ Scored <1.5 SD below the mean of the control group.

### The relationship between deficits to discourse coherence and other language and cognitive components in aphasia

To test the degree to which coherence deficits were attributable to language and/or cognitive components, several regression models were tested, as described in the Methods (see Table 3). The simultaneous multiple regression Model 1 was significant (*F*(3,42) = 25.88, *P* < 0.0001), with language components explaining 65% of the unique variance in discourse coherence, in which phonological production (*B* = 0.132, *P* < 0.0001), and semantic processing (*B* = 0.101, *P* < 0.0001) were the only significant predictors. Adding three connected speech components to the regression equation (Model 2) reached significance (*F*(6,39) = 26.97, *P* < 0.0001) and improved the model fit to explain 81% of the variance in coherence, with verbal fluency presenting as a significant predictor (*B* = 0.08, *P* < 0.0001) in addition to phonological production and semantic processing. Adding the executive functions component to the regression equation (Model 3) did not improve the model fit further (*F*(7,38) = 22.65, *P* < 0.0001), and the executive functions component was not a significant predictor. Finally, adding interaction terms between executive functions and the significant variables (executive functions × phonological production, executive functions × semantic processing, executive functions × verbal fluency) to the regression equation (Model 4) did not improve the model fit, and none of the interaction terms reached significance. We repeated the multiple regression analysis using executive function alone without including any of the language components but found that the Model was not significant and executive function only explained 0.1% of the variance in discourse coherence. These findings (Table 3) indicated that discourse coherence in post-stroke aphasia is strongly related to general language domains including phonological production, verbal fluency, and semantic processing, which explained 81% of the unique variance in coherence; and that domain-general executive functions do not independently relate to coherence in this patient group.

**Table 3:** Language and cognitive components that are attributed to coherence deficits in post-stroke aphasia

Regression	Variables included in the model	Significant variables (*P* < 0.001)	Total variance explained
Model 1	**Phonological production, semantic processing, phonological recognition**	Phonological production, semantic processing	65%
Model 2	Phonological production, semantic processing, phonological recognition, **verbal fluency, verbal quality, motor speech**	Phonological production, semantic processing, verbal fluency	81%
Model 3	Phonological production, semantic processing, phonological recognition, verbal fluency, verbal quality, motor speech, **executive functions**	Phonological production, semantic processing, verbal fluency	81%
Model 4	Phonological production, semantic processing, phonological recognition, verbal fluency, verbal quality, motor speech, executive functions, **executive functions** × **phonological production, executive functions** × **semantic processing, executive functions × verbal fluency**	Phonological production, semantic processing, verbal fluency	84%

Bold represents new variables added to this regression model.

### The lesion correlates associated with discourse coherence deficits

**Figure 2:**
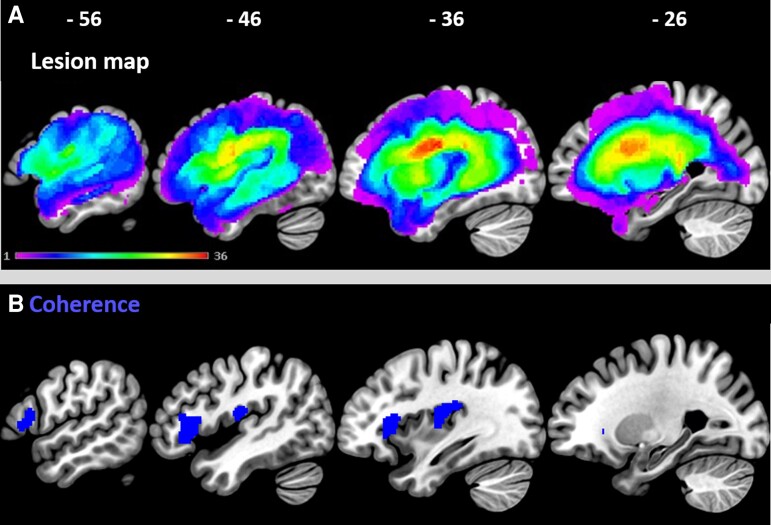
**Lesion overlap map and lesion correlates associated with coherence deficits.** (**A**) Lesion overlap map illustrating the lesion distribution across 46 patients with post-stroke aphasia. The heatmap scale represents the number of patients with a lesion at a given location (hot colours represent more patients and cold colours represent fewer patients). The maximum number of participants who had a lesion in one voxel was 36 (central opercular cortex). (**B**) The neural correlates associated with coherence (blue clusters) identified using VBCM thresholded at *P* < 0.001 voxel-level and FWE cluster-level corrected at *P* < 0.05

The lesion overlap map is illustrated in Fig. 2A. The results from the VBCM (Fig. 2B) revealed two significant clusters associated with coherence deficits while controlling for age and stroke onset, both in the left frontal lobe. The first cluster involved the IFG (pars triangularis and pars opercularis) and the frontal operculum cortex. The second cluster extended medially and involved the insular and central opercular cortex. The model with lesion volume correction did not produce significant clusters. As the behavioural results indicated significant relationships between coherence and other language components (verbal fluency, phonological production, and semantics), we re-ran the analysis and included these language components in the model in addition to discourse coherence. This analysis is interesting to test whether the identified lesion correlates are uniquely associated with coherence deficits or if they are influenced by the patients’ overall language abilities in phonology, verbal fluency and semantics. No areas that were uniquely associated with discourse coherence survived the statistical threshold of this analysis; nor were any of the other language components associated with damage to the IFG. This suggests that the left IFG makes wide-ranging contributions to language processing that cannot be reduced to any single unique aspect of language.

**Figure 3:**
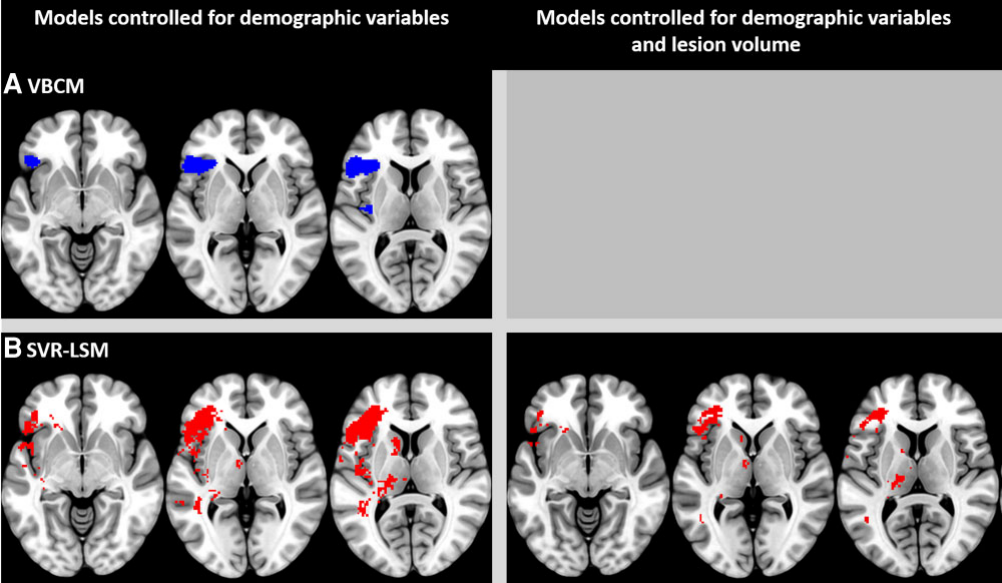
**Neuroimaging results using different lesion-symptom mapping approaches showing the lesion correlates associated with coherence deficits.** MNI coordinates of slices from left to right: Z = −6, 1, 9. (A) VBCM results (blue clusters) thresholded at *P* < 0.001 voxel-wise and FWE cluster-corrected at *P* < 0.05. (B) SVR-LSM results (red clusters) showing the significant beta weights after 100 00 permutation testing, *P* < 0.005 voxel-wise and *P* < 0.05 cluster-wise for the model without lesion volume correction (*left*); and the model with lesion volume correction showing the significant beta weights after 100 00 permutation testing, *P* < 0.05 voxel-wise and *P* < 0.05 cluster-wise (*right*). A grey surface in this figure indicates that no significant results were found for the respective approach

The multivariate analyses yielded similar results to the univariate analyses (see Fig. 3), but the SVR-LSM identified significant clusters associated with coherence deficits while controlling for age and stroke onset that are more widespread, extending posteriorly and medially. The clusters involved the left IFG (pars triangularis and pars opercularis), frontal operculum cortex, frontal orbital cortex, insular cortex and central opercular cortex. When lesion volume was added to the model as a covariate, the SVR-LSM produced significant clusters only at a lenient threshold of *P* < 0.05 voxel-wise and corrected for cluster size at *P* < 0.05, both based on 100 00 permutations. The clusters associated with coherence deficits involved the left IFG (pars triangularis and pars opercularis), frontal operculum cortex, frontal orbital cortex and insular cortex.

## Discussion

This is the first study to provide empirical evidence on the cognitive and neural underpinnings of discourse coherence in post-stroke aphasia across the whole aphasia spectrum, using a computational linguistic measure of coherence. The study provides three main findings. First, people with aphasia exhibited reduced global coherence compared to neuro-typical controls. Second, discourse coherence in people with both fluent and non-fluent aphasia classifications was attributed to deficits in general language domains, including phonological production, verbal fluency and semantic processing; rather than executive functions or motor speech. Finally, coherence deficits are associated with lesions in the left prefrontal cortex. We discuss these findings in the following sub-sections.

### People with aphasia exhibit impaired coherence and this is attributed to deficits in general language domains

Patients with post-stroke aphasia presented with impaired global coherence compared to neuro-typical adults, in that their connected speech was less relevant to the topic under discussion. These results are in line with previous studies reporting impaired global coherence in people with brain damage, including those with right hemispheric stroke,^4^ traumatic brain injury,^7,8^ post-stroke aphasia^9–12,14^ and Alzheimer’s disease.^7^ Our findings extend the previous literature by showing that impaired coherence in a large group of patients with various classifications of fluent and non-fluent aphasia can be attributed to their performance on core language components. Specifically, lower global coherence was associated with deficits in phonological production and semantic processing, and with reduced fluency. We also found that measurements of discourse coherence can be influenced by the discourse task. The current results showed that a tightly-constrained discourse topic, such as that used in a procedural task, can generate high measurements of global coherence in both healthy people and patients, compared to descriptive or storytelling tasks. This is probably because descriptive and storytelling tasks require a wider range of themes and concepts to be discussed, which necessarily causes utterances to move further away from the central prototype we used to assess coherence. This highlights the importance of carefully choosing the discourse task to address the specific research or clinical aims and of ensuring that all participants are assessed using the same type of discourse task.

Impairments in phonological production involve word retrieval deficits that can lead to reduced global coherence, due to the associated word retrieval errors (e.g. circumlocutions, paraphasias and fillers) that might disrupt the flow of speech and result in production of speech that is not related to the topic under discussion. An association between word retrieval deficits and higher production of global coherence errors has been reported in anomic aphasia.^10^ Other studies have found reduced lexical diversity,^12^ word retrieval errors and reduced information content^14^ to contribute to lower coherence in fluent aphasia. Our results extend these findings by showing an association between phonological production and coherence across a wide range of aphasia classifications beyond fluent and anomic aphasia.

Impaired semantic processing may also lead to production of semantic paraphasias, which, in turn, can reduce global coherence if these paraphasias were unrelated to the topic under discussion. This has been observed in people with schizophrenia, whose global coherence errors were associated with semantic errors.^6^ An association between poor performance on synonym tests that tap into semantic knowledge and increased global coherence errors has also been reported in right hemisphere stroke patients.^4^ To maintain coherent speech, it is necessary to select topic-relevant ideas and semantic information from the multiple semantically related competitors that become activated in response to a speech stimulus. This selection process is thought to be governed via semantic control processes that regulate access to semantic knowledge.^16,17^ A recent case-series study revealed a strong correlation between deficits in semantic control and impaired coherence in patients with post-stroke semantic deficits.^17^ The authors suggested that their findings highlighted the role of semantic control in regulating coherent speech production. The correlation we found in this study between impaired coherence and semantic processing could reflect a combination of the effects of the presence of semantic paraphasias and deficits in semantic control.

In contrast, we observed no association between discourse coherence and general executive functions or the interaction between semantic processing and executive functions. This suggests that in the context of post-stroke aphasia and among a broader sample including those with fluent and non-fluent classifications, there is no effect of general executive functions on discourse coherence, beyond the contribution of semantic control processes (though of course it remains possible that small effects could be detected in a larger group of patients). On the other hand, general executive mechanisms do seem to play a greater role in discourse coherence in other clinical groups where executive deficits are more prominent and central than they typically are in post-stroke aphasia.^4,8^ This might include patients with lesions to the multiple demand network^56^ who present with impaired executive functions, rather than those who present with a predominant language deficit due to stroke lesions mainly within the middle cerebral artery territory. This hypothesis is supported by findings from non-aphasic stroke patients with mild cognitive deficits, whose better performance on executive function and attention tasks was related to reduced global coherence errors.^4^ We only included patients who developed aphasia post-stroke in this study, and thus discourse coherence could be influenced by other aspects of executive functions, including attention, in healthy people or in other patient groups.

The association between reduced fluency and low coherence in connected speech is novel to this study. This is perhaps because previous aphasiological studies that addressed coherence included patients with fluent aphasia and/or mild to moderate deficits,^4,10–12,17^ whereas we also included non-fluent and severe patients in our study. This association is, however, understandable given that reduced fluency might lead to the production of perseverative, repetitive and filler utterances, and these irrelevant words and utterances might reduce the level of global coherence. In summary, our findings indicate that difficulties in the ability to construct and maintain coherent discourse are related to core language deficits in phonology, fluency and semantics in a heterogeneous group of people with post-stroke aphasia ranging from mild anomia to severe non-fluent aphasia.

### Discourse coherence is supported by the left prefrontal cortex

The lesion correlates of discourse coherence were explored using univariate and multivariate lesion-symptom mapping approaches. The analyses indicated that lesions in the left prefrontal cortex were associated with coherence deficits. Specifically, the IFG (pars triangularis, pars opercularis), frontal operculum cortex, central opercular cortex, frontal orbital cortex and insular cortex were identified as lesion correlates using both univariate and multivariate approaches. Whilst this is the first study to use lesion analysis in identifying the lesion correlates of coherence deficits, the results are convergent with those of an fMRI study showing increased activation in the IFG (pars triangularis) when healthy older adults produced highly coherent speech;^29^ and with a neuro-stimulation experiment indicating that stimulation to the left IFG in healthy participants increased global coherence errors.^33^ This converging evidence suggests a role for the left IFG (pars triangularis) in producing coherent discourse. This region has been implicated in previous neuroimaging studies in selecting task-relevant semantic knowledge and episodic memories,^30,57,58^ which is consistent with the idea that producing coherent discourse relies on appropriate access to both general semantic knowledge and episodic memories related to specific experiences and events. The IFG may play an important role in the regulation of connected speech output, by ensuring that context-relevant concepts are selected for use in discourse, and by inhibiting irrelevant thoughts that come to mind. Left frontal regions contribute to the regulation of language processing more generally, including for the domains of fluency, phonology and semantics, thus damage to this region could cause wide-ranging language deficits beyond impairment to coherence. This would also converge with our behavioural results, in that coherence in post-stroke aphasia seems to be related to general language abilities.

## Conclusions

The novel contribution of this study was to provide insights on the cognitive and neural underpinnings of discourse coherence in post-stroke aphasia. Deficits in maintaining coherent discourse can be attributed to impairments in core language components, including phonological production, verbal fluency and semantic processing, but not general executive functions. Impaired discourse coherence was associated with damage to left prefrontal cortex, including the IFG (pars triangularis and pars opercularis). This result adds to converging evidence that this region plays an important role in regulating connected speech production and selecting topic-relevant aspects of knowledge. Our results provide empirical bases for the understanding of the root cognitive and neural causes of coherence deficits in people with aphasia. This knowledge can lead to better clinical assessment and management, which is valuable given the critical role of coherent discourse in conversation.

## Abbreviations

IFG =: inferior frontal gyrus
LSA =: latent semantic analysis
SVR-LSM =: support-vector regression lesion-symptom mapping
VBCM =: voxel-based correlational methodology
